# *N*′-(5-Bromo-2-hydr­oxy-3-methoxy­benzyl­idene)isonicotinohydrazide

**DOI:** 10.1107/S1600536808029607

**Published:** 2008-09-24

**Authors:** San-Jun Peng, Hai-Yun Hou

**Affiliations:** a College of Chemistry and Biological Engineering, Changsha University of Science and Technology, Changsha 410076, People’s Republic of China; b College of Environmental and Chemical Engineering, Xi’an Polytechnic University, Xi’an 710048, People’s Republic of China

## Abstract

The title compound, C_14_H_12_BrN_3_O_3_, was prepared by reaction of 5-bromo-3-methoxy­salicylaldehyde and isonicotinohydrazide in methanol. The mol­ecule is not planar and adopts a *trans* configuration with respect to the C=N bond. There is an intra­molecular O—H⋯N hydrogen bond in the mol­ecule. The dihedral angle between the benzene and pyridine rings is 12.2 (2)°. In the crystal structure, mol­ecules are linked through inter­molecular N—H⋯N hydrogen bonds, forming chains running along the *c*-axis direction.

## Related literature

For bond-length data, see: Allen *et al.* (1987 ▶). For background on the biological properties of hydrazones, see: El-Tabl *et al.* (2008 ▶), Chen *et al.* (2008 ▶); Alvarez *et al.* (2008 ▶); Ventura & Martins (2008 ▶); Kalinowski *et al.* (2008 ▶). For related structures, see: Peng & Hou (2008 ▶); Shan *et al.* (2008 ▶); Fun *et al.* (2008 ▶); Yehye *et al.* (2008 ▶); Ejsmont *et al.* (2008 ▶); Han *et al.* (2006 ▶); Lu *et al.* (2008 ▶).
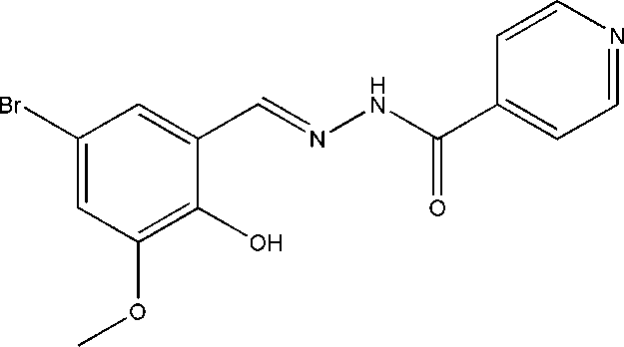


## Experimental

### 

#### Crystal data

C_14_H_12_BrN_3_O_3_
*M* *_r_* = 350.18
Monoclinic, 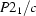

*a* = 7.4937 (9) Å
*b* = 15.8843 (19) Å
*c* = 11.7994 (14) Å
β = 99.776 (2)°
*V* = 1384.1 (3) Å^3^
*Z* = 4
Mo *K*α radiation
μ = 2.98 mm^−1^
*T* = 298 (2) K
0.20 × 0.18 × 0.18 mm

#### Data collection

Bruker SMART 1000 CCD area-detector diffractometer
Absorption correction: multi-scan (*SADABS*; Bruker, 2001 ▶) *T*_min_ = 0.587, *T*_max_ = 0.616 (expected range = 0.557–0.584)
8003 measured reflections
3013 independent reflections
2299 reflections with *I* > 2σ(*I*)
*R*_int_ = 0.023

#### Refinement

*R*[*F* ^2^ > 2σ(*F* ^2^)] = 0.030
*wR*(*F* ^2^) = 0.076
*S* = 1.03
3013 reflections
195 parameters
1 restraint
H atoms treated by a mixture of independent and constrained refinement
Δρ_max_ = 0.39 e Å^−3^
Δρ_min_ = −0.41 e Å^−3^

### 

Data collection: *SMART* (Bruker, 2007 ▶); cell refinement: *SAINT* (Bruker, 2007 ▶); data reduction: *SAINT*; program(s) used to solve structure: *SHELXTL* (Sheldrick, 2008 ▶); program(s) used to refine structure: *SHELXTL*; molecular graphics: *SHELXTL*; software used to prepare material for publication: *SHELXTL*.

## Supplementary Material

Crystal structure: contains datablocks global, I. DOI: 10.1107/S1600536808029607/sj2539sup1.cif

Structure factors: contains datablocks I. DOI: 10.1107/S1600536808029607/sj2539Isup2.hkl

Additional supplementary materials:  crystallographic information; 3D view; checkCIF report

## Figures and Tables

**Table 1:** Hydrogen-bond geometry (Å, °)

*D*—H⋯*A*	*D*—H	H⋯*A*	*D*⋯*A*	*D*—H⋯*A*
N2—H2⋯N3^i^	0.889 (10)	2.255 (13)	3.126 (3)	166 (3)
O1—H1⋯N1	0.82	1.93	2.643 (2)	145

Symmetry code: (i) 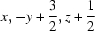.

**Fig. 1.**
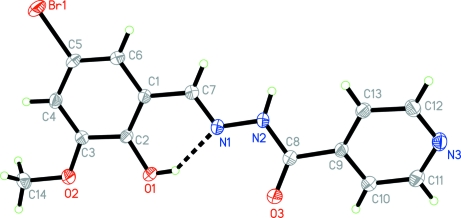
The molecular structure of the title compound with 30% probability displacement ellipsoids for non-H atoms; the intramolecular hydrogen bond is drawn as a dashed line.

**Fig. 2.**
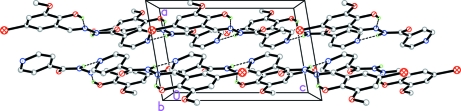
The packing diagram of the title compound, viewed down the b axis. Hydrogen bonds are shown as dashed lines and hydrogen atoms not involved in these interactions have been omitted..

## supplementary crystallographic information

### Comment

Hydrazones derived from the reactions of aldehydes with hydrazides show potential biological properties (El-Tabl *et al.*, 2008; Chen *et al.*, 2008; Alvarez *et al.*, 2008; Ventura & Martins, 2008; Kalinowski *et al.*, 2008). In the last few years, a large number of hydrazones have been reported (Peng & Hou, 2008; Shan *et al.*, 2008; Fun *et al.*, 2008; Yehye *et al.*, 2008; Ejsmont *et al.*, 2008). As a continuation of our work in this area (Peng & Hou, 2008) we report here the crystal structure of the title compound, (I), Fig. 1.

In the molecule of the title compound (I) the C7═N1  length of 1.275 (3) Å indicates a typical C═N bond. The molecule exists in a *trans* configuration with respect to the methylidene unit (C7═N1), as observed in other similar compounds (Han *et al.*, 2006; Lu *et al.*, 2008). There is an intramolecular O—H···N hydrogen bond in the molecule. The dihedral angle between the benzene and pyridine rings is 12.2 (2)°, indicating the molecule is not planar. The bond lengths are in normal ranges (Allen *et al.*, 1987).

In the crystal structure, molecules are linked through intermolecular N—H···N hydrogen bonds (Table 1), forming chains running along the c direction (Fig. 2).

### Experimental

5-Bromo-3-methoxysalicylaldehyde (0.231 g, 1 mmol) was dissolved in methanol (50 ml), then isonicotinohydrazide (0.137 g, 1 mmol) was added slowly to the solution, and the mixture was heated at reflux with continuous stirring for 1 h. The solution was cooled to room temperature, yielding colorless crystallites. Recrystallization from an absolute methanol yielded block-like single crystals of the compound.

### Refinement

H2 was located in a difference Fourier map and refined isotropically, with the N—H distance restrained to 0.90 (1) Å, and with *U*_iso_ set at 0.08 Å^2^. Other H atoms were placed in calculated positions with C—H distances of 0.93–0.96 Å, O—H distance of 0.82 Å, and refined in riding mode with *U*_iso_(H) = 1.2*U*_eq_(C) and 1.5*U*_eq_(O).

### Crystal data

**Table:**

C_14_H_12_BrN_3_O_3_	*F*(000) = 704
*M**_r_* = 350.18	*D*_x_ = 1.680 Mg m^−^^3^
Monoclinic, *P*2_1_/*c*	Mo *K*α radiation, λ = 0.71073 Å
Hall symbol: -P 2ybc	Cell parameters from 3223 reflections
*a* = 7.4937 (9) Å	θ = 2.5–29.2°
*b* = 15.8843 (19) Å	µ = 2.98 mm^−^^1^
*c* = 11.7994 (14) Å	*T* = 298 K
β = 99.776 (2)°	Block, colorless
*V* = 1384.1 (3) Å^3^	0.20 × 0.18 × 0.18 mm
*Z* = 4	

### Data collection

**Table:**

Bruker SMART 1000 CCD area-detector diffractometer	3013 independent reflections
Radiation source: fine-focus sealed tube	2299 reflections with *I* > 2σ(*I*)
graphite	*R*_int_ = 0.023
ω scans	θ_max_ = 27.0°, θ_min_ = 2.6°
Absorption correction: multi-scan (SADABS; Bruker, 2001)	*h* = −9→9
*T*_min_ = 0.587, *T*_max_ = 0.616	*k* = −17→20
8003 measured reflections	*l* = −15→11

### Refinement

**Table:**

Refinement on *F*^2^	Primary atom site location: structure-invariant direct methods
Least-squares matrix: full	Secondary atom site location: difference Fourier map
*R*[*F*^2^ > 2σ(*F*^2^)] = 0.030	Hydrogen site location: inferred from neighbouring sites
*wR*(*F*^2^) = 0.076	H atoms treated by a mixture of independent and constrained refinement
*S* = 1.03	*w* = 1/[σ^2^(*F*_o_^2^) + (0.0328*P*)^2^ + 0.5984*P*] where *P* = (*F*_o_^2^ + 2*F*_c_^2^)/3
3013 reflections	(Δ/σ)_max_ = 0.001
195 parameters	Δρ_max_ = 0.39 e Å^−^^3^
1 restraint	Δρ_min_ = −0.41 e Å^−^^3^

### Special details

**Table:**

Geometry. All e.s.d.'s (except the e.s.d. in the dihedral angle between two l.s. planes) are estimated using the full covariance matrix. The cell e.s.d.'s are taken into account individually in the estimation of e.s.d.'s in distances, angles and torsion angles; correlations between e.s.d.'s in cell parameters are only used when they are defined by crystal symmetry. An approximate (isotropic) treatment of cell e.s.d.'s is used for estimating e.s.d.'s involving l.s. planes.
Refinement. Refinement of *F*^2^ against ALL reflections. The weighted *R*-factor *wR* and goodness of fit *S* are based on *F*^2^, conventional *R*-factors *R* are based on *F*, with *F* set to zero for negative *F*^2^. The threshold expression of *F*^2^ > σ(*F*^2^) is used only for calculating *R*-factors(gt) *etc*. and is not relevant to the choice of reflections for refinement. *R*-factors based on *F*^2^ are statistically about twice as large as those based on *F*, and *R*- factors based on ALL data will be even larger.

### Fractional atomic coordinates and isotropic or equivalent isotropic displacement parameters (Å^2^)

**Table:**

	*x*	*y*	*z*	*U*_iso_*/*U*_eq_	
Br1	0.28353 (5)	0.417081 (18)	1.029574 (19)	0.06245 (12)	
O1	0.1144 (2)	0.35958 (10)	0.51873 (12)	0.0457 (4)	
H1	0.1510	0.3985	0.4832	0.069*	
O2	0.0357 (2)	0.24186 (10)	0.65725 (13)	0.0473 (4)	
O3	0.2696 (3)	0.49630 (11)	0.27412 (14)	0.0651 (6)	
N1	0.2592 (2)	0.50906 (11)	0.49603 (15)	0.0366 (4)	
N2	0.3058 (3)	0.57618 (11)	0.43355 (15)	0.0368 (4)	
N3	0.3899 (3)	0.77447 (12)	0.10697 (16)	0.0422 (5)	
C1	0.2305 (3)	0.45105 (13)	0.67791 (17)	0.0322 (5)	
C2	0.1524 (3)	0.37588 (13)	0.63313 (16)	0.0314 (4)	
C3	0.1127 (3)	0.31267 (13)	0.70889 (17)	0.0328 (5)	
C4	0.1548 (3)	0.32447 (14)	0.82630 (17)	0.0352 (5)	
H4	0.1314	0.2823	0.8765	0.042*	
C5	0.2323 (3)	0.39986 (14)	0.86841 (17)	0.0370 (5)	
C6	0.2695 (3)	0.46250 (14)	0.79697 (17)	0.0375 (5)	
H6	0.3208	0.5127	0.8274	0.045*	
C7	0.2776 (3)	0.51825 (13)	0.60474 (18)	0.0376 (5)	
H7	0.3220	0.5689	0.6378	0.045*	
C8	0.3013 (3)	0.56400 (14)	0.31958 (18)	0.0379 (5)	
C9	0.3367 (3)	0.64012 (13)	0.25079 (17)	0.0321 (5)	
C10	0.2656 (3)	0.63897 (14)	0.13450 (18)	0.0408 (5)	
H10	0.1991	0.5929	0.1022	0.049*	
C11	0.2945 (3)	0.70697 (15)	0.0670 (2)	0.0449 (6)	
H11	0.2445	0.7055	−0.0107	0.054*	
C12	0.4597 (3)	0.77448 (14)	0.2190 (2)	0.0395 (5)	
H12	0.5287	0.8207	0.2484	0.047*	
C13	0.4358 (3)	0.71018 (13)	0.29379 (18)	0.0350 (5)	
H13	0.4853	0.7138	0.3714	0.042*	
C14	0.0108 (3)	0.17267 (14)	0.7293 (2)	0.0473 (6)	
H14A	0.1255	0.1566	0.7732	0.071*	
H14B	−0.0397	0.1261	0.6828	0.071*	
H14C	−0.0701	0.1886	0.7805	0.071*	
H2	0.331 (4)	0.6240 (12)	0.472 (2)	0.080*	

### Atomic displacement parameters (Å^2^)

**Table:**

	*U*^11^	*U*^22^	*U*^33^	*U*^12^	*U*^13^	*U*^23^
Br1	0.1005 (3)	0.0644 (2)	0.02233 (13)	−0.01755 (16)	0.01035 (12)	−0.00145 (11)
O1	0.0749 (12)	0.0384 (9)	0.0233 (7)	−0.0087 (8)	0.0068 (7)	−0.0007 (6)
O2	0.0711 (11)	0.0338 (8)	0.0357 (8)	−0.0121 (8)	0.0053 (8)	0.0022 (7)
O3	0.1248 (17)	0.0381 (9)	0.0351 (9)	−0.0254 (10)	0.0207 (10)	−0.0054 (8)
N1	0.0502 (12)	0.0317 (10)	0.0292 (9)	0.0001 (8)	0.0104 (8)	0.0049 (7)
N2	0.0560 (12)	0.0280 (9)	0.0282 (9)	−0.0011 (9)	0.0123 (8)	0.0047 (7)
N3	0.0509 (12)	0.0379 (10)	0.0394 (10)	0.0030 (9)	0.0122 (9)	0.0087 (8)
C1	0.0411 (12)	0.0296 (10)	0.0269 (10)	0.0031 (9)	0.0082 (9)	0.0025 (8)
C2	0.0386 (12)	0.0318 (11)	0.0234 (9)	0.0049 (9)	0.0043 (8)	0.0006 (8)
C3	0.0373 (12)	0.0300 (11)	0.0313 (10)	0.0017 (9)	0.0062 (9)	0.0005 (9)
C4	0.0412 (13)	0.0364 (12)	0.0296 (10)	0.0030 (10)	0.0105 (9)	0.0059 (9)
C5	0.0477 (13)	0.0427 (13)	0.0215 (10)	−0.0004 (10)	0.0078 (9)	−0.0016 (9)
C6	0.0511 (14)	0.0339 (11)	0.0279 (10)	−0.0022 (10)	0.0079 (9)	−0.0036 (9)
C7	0.0523 (14)	0.0302 (11)	0.0309 (11)	−0.0003 (10)	0.0089 (10)	0.0000 (9)
C8	0.0502 (14)	0.0349 (12)	0.0296 (10)	−0.0024 (10)	0.0100 (10)	0.0013 (9)
C9	0.0390 (12)	0.0307 (11)	0.0290 (10)	0.0036 (9)	0.0123 (9)	0.0016 (8)
C10	0.0545 (15)	0.0368 (12)	0.0306 (11)	−0.0033 (11)	0.0059 (10)	−0.0002 (9)
C11	0.0582 (16)	0.0452 (14)	0.0301 (11)	0.0021 (12)	0.0042 (10)	0.0042 (10)
C12	0.0425 (13)	0.0334 (12)	0.0437 (12)	−0.0004 (10)	0.0108 (10)	0.0014 (10)
C13	0.0401 (12)	0.0361 (12)	0.0295 (10)	0.0031 (9)	0.0080 (9)	0.0001 (9)
C14	0.0624 (16)	0.0335 (12)	0.0482 (14)	−0.0057 (11)	0.0158 (12)	0.0047 (11)

### Geometric parameters (Å, °)

**Table:**

Br1—C5	1.895 (2)	C4—C5	1.386 (3)
O1—C2	1.356 (2)	C4—H4	0.9300
O1—H1	0.8200	C5—C6	1.363 (3)
O2—C3	1.360 (3)	C6—H6	0.9300
O2—C14	1.421 (3)	C7—H7	0.9300
O3—C8	1.207 (3)	C8—C9	1.505 (3)
N1—C7	1.275 (3)	C9—C10	1.385 (3)
N1—N2	1.375 (2)	C9—C13	1.386 (3)
N2—C8	1.353 (3)	C10—C11	1.381 (3)
N2—H2	0.889 (10)	C10—H10	0.9300
N3—C11	1.330 (3)	C11—H11	0.9300
N3—C12	1.336 (3)	C12—C13	1.381 (3)
C1—C2	1.394 (3)	C12—H12	0.9300
C1—C6	1.397 (3)	C13—H13	0.9300
C1—C7	1.454 (3)	C14—H14A	0.9600
C2—C3	1.409 (3)	C14—H14B	0.9600
C3—C4	1.380 (3)	C14—H14C	0.9600
			
C2—O1—H1	109.5	N1—C7—H7	119.4
C3—O2—C14	117.41 (17)	C1—C7—H7	119.4
C7—N1—N2	117.18 (18)	O3—C8—N2	122.6 (2)
C8—N2—N1	117.15 (18)	O3—C8—C9	121.04 (19)
C8—N2—H2	127 (2)	N2—C8—C9	116.37 (18)
N1—N2—H2	116 (2)	C10—C9—C13	117.73 (19)
C11—N3—C12	116.57 (19)	C10—C9—C8	116.78 (19)
C2—C1—C6	119.70 (19)	C13—C9—C8	125.48 (19)
C2—C1—C7	122.19 (18)	C11—C10—C9	119.3 (2)
C6—C1—C7	118.10 (19)	C11—C10—H10	120.4
O1—C2—C1	122.99 (18)	C9—C10—H10	120.4
O1—C2—C3	117.65 (18)	N3—C11—C10	123.6 (2)
C1—C2—C3	119.35 (18)	N3—C11—H11	118.2
O2—C3—C4	124.68 (19)	C10—C11—H11	118.2
O2—C3—C2	115.10 (18)	N3—C12—C13	124.1 (2)
C4—C3—C2	120.20 (19)	N3—C12—H12	118.0
C3—C4—C5	119.18 (19)	C13—C12—H12	118.0
C3—C4—H4	120.4	C12—C13—C9	118.7 (2)
C5—C4—H4	120.4	C12—C13—H13	120.7
C6—C5—C4	121.76 (19)	C9—C13—H13	120.7
C6—C5—Br1	119.16 (16)	O2—C14—H14A	109.5
C4—C5—Br1	119.06 (16)	O2—C14—H14B	109.5
C5—C6—C1	119.8 (2)	H14A—C14—H14B	109.5
C5—C6—H6	120.1	O2—C14—H14C	109.5
C1—C6—H6	120.1	H14A—C14—H14C	109.5
N1—C7—C1	121.2 (2)	H14B—C14—H14C	109.5

### Hydrogen-bond geometry (Å, °)

**Table:**

*D*—H···*A*	*D*—H	H···*A*	*D*···*A*	*D*—H···*A*
N2—H2···N3^i^	0.89 (1)	2.26 (1)	3.126 (3)	166 (3)
O1—H1···N1	0.82	1.93	2.643 (2)	145.

Symmetry codes: (i) *x*, −*y*+3/2, *z*+1/2.
